# Supplementary figures and images for: The quarantine hospital strategy as a way to reduce both community and nosocomial transmission in the context of a COVID-like epidemic
Source: PLoS Comput Biol. 2025 Oct 3;21(10):e1013548. doi: 10.1371/journal.pcbi.1013548 (PMC12507276; doi:10.1371/journal.pcbi.1013548)

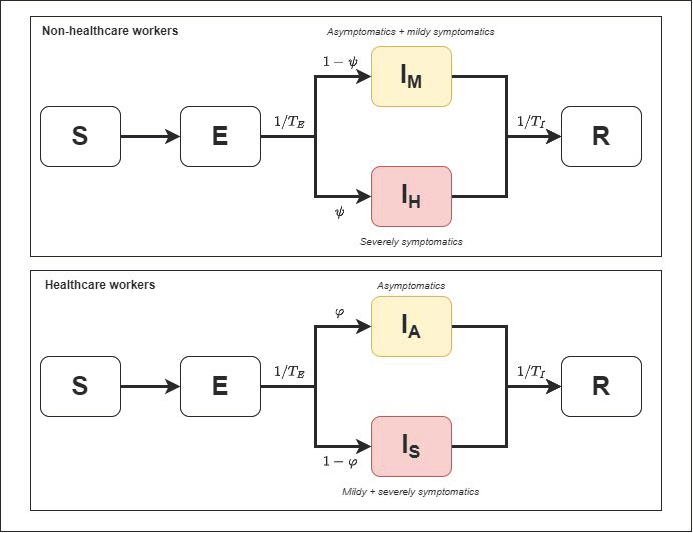

Supplement: S1 Fig — Diagram describing successive infections statuses for non-HCWs (upper panel) and HCWs (lower panel). (TIFF) [file pcbi.1013548.s001.tif]

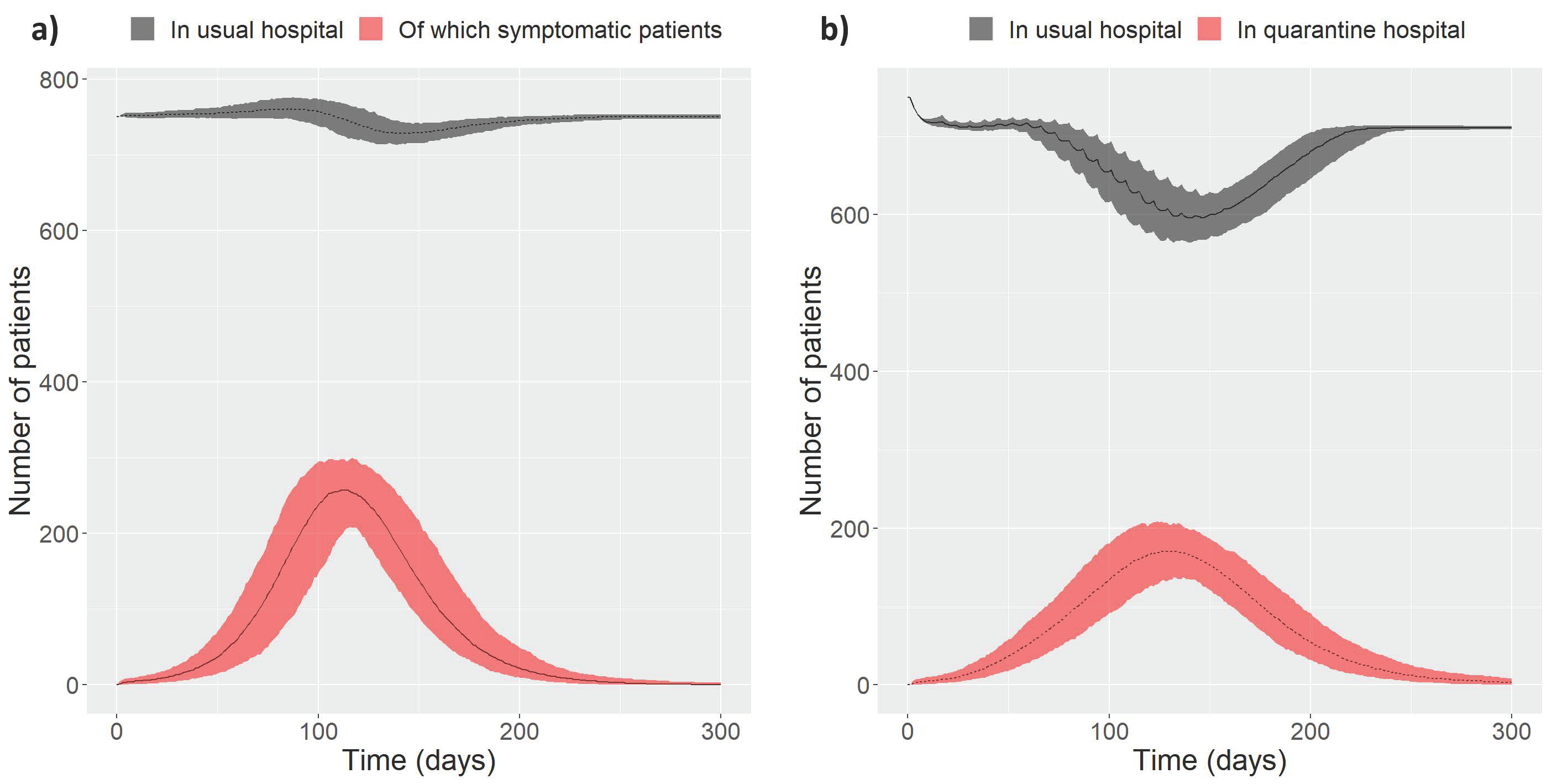

Supplement: S2 Fig — Median number of patient admissions, along with the 95% prediction band, as a function of time (a) in the reference strategy (all admissions to the usual hospital in black, admissions of symptomatic patients to the usual hospital in red); and (b) in the quarantine strategy (all admissions to the usual hospital in black, all admissions to the quarantine hospital in red). (TIFF) [file pcbi.1013548.s002.tif]

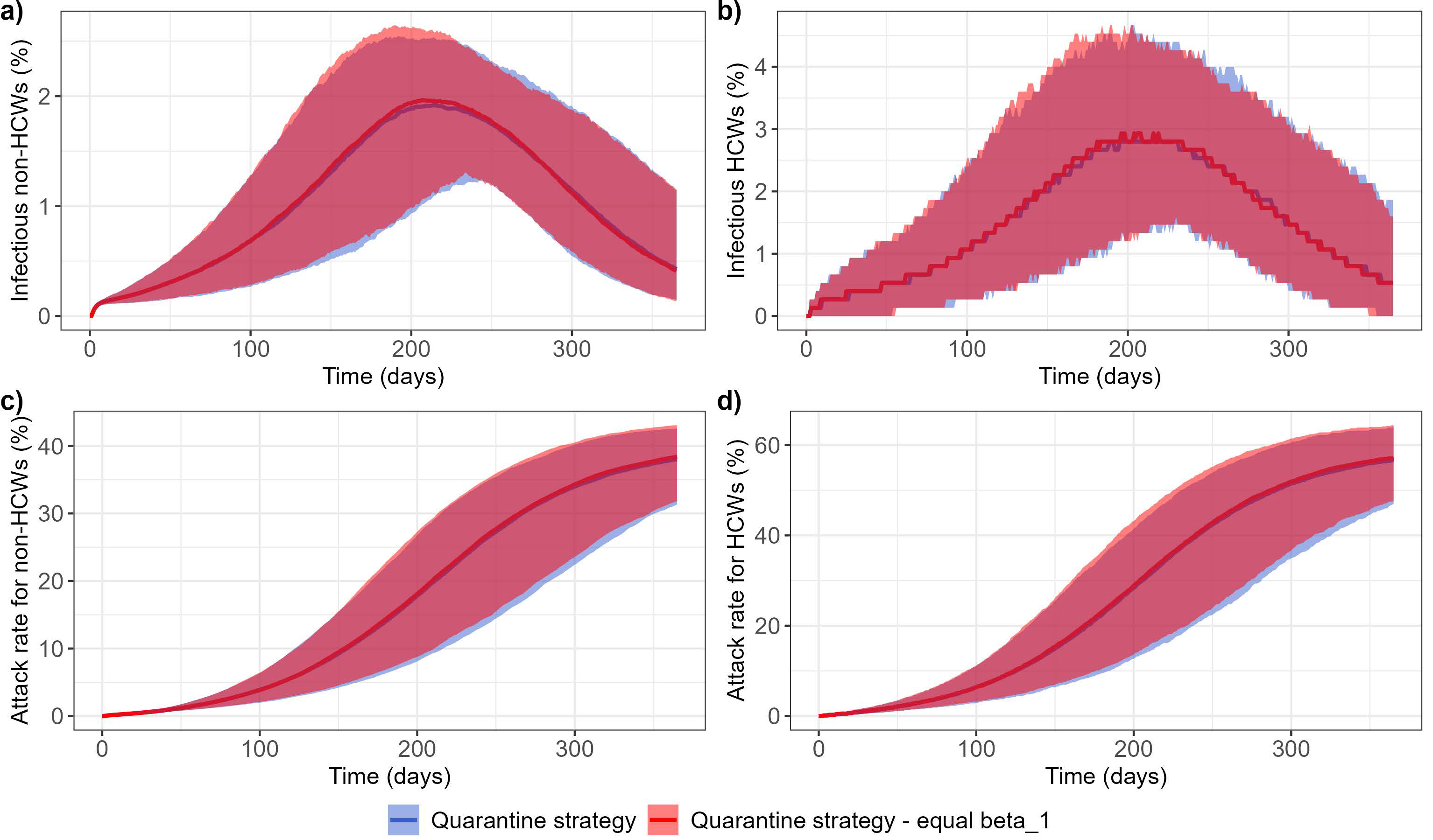

Supplement: S3 Fig — Predicted changes over 365 days in the median and 95% prediction band of the percentage of infectious individuals (I and A compartments) amongst a) non-healthcare workers and b) healthcare workers. Attack rate in c) non-healthcare workers and d) healthcare workers. (TIFF) [file pcbi.1013548.s003.tif]
